# *Toxoplasma gondii* in meat of adult sheep in Spain

**DOI:** 10.1016/j.fawpar.2023.e00203

**Published:** 2023-06-30

**Authors:** María Paz Peris, Amalia Xía García, Juan Antonio Castillo, Juan José Badiola, Nabil Halaihel, María Serrano, María Jesús Gracia

**Affiliations:** Animal Pathology Department, Faculty of Veterinary Sciences, University of Zaragoza, Spain

**Keywords:** *Toxoplasma gondii*, Adult sheep, Meat, Sheep meat, Real-time PCR, Spain

## Abstract

Toxoplasmosis is a zoonotic disease caused by *Toxoplasma gondii*, an intracellular parasite that presents a worldwide risk. Humans can become infected by ingesting meat infected with *T. gondii*, and the consumption of infected sheep and goat meat is a significant public health issue. Antibodies against *T. gondii* have been found in sheep in Spain, indicating the presence of the parasite in the country. However, no previous studies have assessed the presence of *T. gondii* in sheep meat in Spain. In view of the significance of the transmission of *T. gondii* through meat consumption and given the lack of previous studies in Spain, we carried out an investigation to evaluate the presence of *T. gondii* in adult sheep meat (mutton). A total of 216 muscle samples were analyzed by digestion, and a real-time PCR assay was used to determine the presence of *T. gondii* DNA. A total of 24.5% of the samples were found to be parasitized, indicating that the consumption of sheep meat can present an important risk for human health.

## Highlights

• *Toxoplasma gondii* was analyzed in adult sheep meat for the first time in Spain.
• *T. gondii* DNA presence in meat was assessed by real-time PCR.
• Of 216 samples, 24.5% were positive for *Toxoplasma gondii.*
• Sheep meat could be an important risk for human health.

## 1 Introduction

*Toxoplasma gondii* is a widespread apicomplexan parasite that causes toxoplasmosis in warm-blooded animals and humans. It is estimated that 25–30% of the world population is seropositive for *T. gondii* (Robert-Gangneux, 2014). Immunocompetent individuals infected with *T. gondii* rarely experience clinical disease. Most infections are subclinical and only 20% of infected humans show mild or more pronounced clinical symptoms and signs (Robert-Gangneux et al., 2015; Montoya et al., 2019). Humans mainly become infected by ingesting raw or undercooked mammalian or poultry meat containing cysts with bradyzoites, as well as through ingestion of food or water contaminated with sporulated oocysts (Montoya et al., 2019).

Serum antibodies against *T. gondii* have been found in sheep worldwide. For example, seroepidemiological surveys in Europe have reported a global distribution ranging from under 25% to over 80% (Klun et al., 2006; Olsen et al., 2019; Tagel et al., 2019). In the case of Spain, a review conducted by Dubey et al. (2020) indicates seroprevalence ranging from 41% to 62% (García-Bocanegra et al., 2013; Díaz et al., 2016; Almería et al., 2018).

A review conducted by Belluco et al. (2016) assessed the presence of *T. gondii* in sheep meat on a global scale: the continents with the highest ratio were Asia, Oceania, and Africa, with a prevalence of 27%, 25%, and 20%, respectively, followed by North America (14%), Europe (9%) and South America (2%). Studies in several different European countries report a prevalence rate ranging from 3.3% to 42% (Jackson et al., 1987; Halos et al., 2010; Mason et al., 2010; Berger-Schoch et al., 2011; Halová et al., 2012; Lopes et al., 2015; Gazzonis et al., 2020). Despite the high seroprevalence detected in sheep in Spain, no study has investigated the presence of *T. gondii* in sheep meat in this country. The consumption of infected meat is generally regarded as an issue of major public health importance, and sheep meat is a significant risk factor (Dardé and Peyron, 2013). In this context and given the importance of the transmission of *T. gondii* through meat consumption and the lack of studies in Spain, we performed the current study to evaluate the presence of *T. gondii* in mutton by real-time PCR.

## 2 Material and methods

### 2.1 Sample selection

The muscle samples used in the present study were collected from the carcasses of sheep slaughtered according to EU Regulation 2017/625 and Spanish RD 640/2006 at an abattoir in Tomelloso (Ciudad Real, Castilla-La Mancha, Spain). Samples were collected by an officially (certified) veterinarian twice a week at different times between May 2019 and February 2020 to ensure that the samples did not stem from the same farm. The slaughterhouse's supply area is the center, west, and south of Spain: specifically, Ciudad Real, Albacete, and Toledo (Castilla-La Mancha), Cáceres and Badajoz (Extremadura), and Jaén and Córdoba (Andalucía), on farms where livestock production is primarily extensive.

A total of 216 samples from female adult sheep (∼2–4 years old), were used for this study. Each sample consisted of two or three fragments from different muscular locations (costal, hind leg, abdomen, armpit, and foreleg). The samples were preserved by refrigeration and immediately transported to the Parasitology Laboratory of the Pathology Department of the Faculty of Veterinary Medicine of the University of Zaragoza (Spain) for examination, kept there at 4 °C and stored for a maximum of 48 h until processing.

### 2.2 Sample preparation

All received meat samples were homogenized and crushed with a pestle and mortar. After mixing, ten grams were extracted, and a concentration technique with an acid pepsin digestion procedure was applied as described by Dubey (1998), with some modifications (Bayarri et al., 2010). Briefly, the ten grams of meat were minced and homogenized with 20 mL of NaCl (0.85%) with a mortar. Then, 20 mL of pepsin solution (pH = 1.1–1.2) was added and the sample was incubated at 37 °C for 30 min under agitation. After digestion, the content was filtered, centrifuged, and added with 25 mL of NaHCO_3_ solution (1.2%, pH = 8.3) to neutralize the pepsin with the aim of preventing the destruction of the parasite. Finally, after two PBS washes, the content was reconstituted in 2 mL of PBS. All samples were processed under the same conditions, and meticulous equipment cleaning and disinfection were carried out while preparing the samples.

### 2.3 Evaluation with real-time quantitative polymerase chain reaction (qPCR)

DNA extraction and the qPCR protocol were performed according to procedures detailed in previous studies (Gracia et al., 2021) with some modifications. Genomic DNA was extracted from a 200 μL volume of the concentrated samples using a commercial Speedtools DNA extraction kit (Biotools B&M Labs S.A, Madrid, Spain). Briefly, 300 μL of lysis buffer was added and homogenized using a pestle with rotating plastic plungers in an Eppendorf tube. After this, the manufacturer's instructions were followed.

For DNA amplification, two sets of primers were used; ToxoRoc and ToxoRepeat 500 (Table 1), targeting the specific sequence of a 529 bp repeat element. The qPCR assay was designed to be amplified by the fluorochrome known as Sybr Green and each reaction was carried out in a final volume of 20 μL containing 10 μL of Sybr Green master mix (GoTaq® Hot Start Green Master Mix 2, Promega, Madison, WI, USA) and 2.5 μL of DNA template. The forward and reverse primer concentration was adjusted to 0.4 mol L^−1^ each.

**Table 1:** Primers used for *Toxoplasma gondii* detection (adopted from Gracia et al., 2021).

Name	Sequences	Length (nt)
ToxoRoc F	5′-TAGACGAGACGACGCTTTCC-3′	64
ToxoRoc R	5′- TCGCCCTCTTCTCCACTCT-3′	
ToxoRepeat 500 F	5′-CGCTGCAGGGAGGAAGACGAAAGTTG-3′	529
ToxoRepeat 500 R	5′-CGCTGCAGACACAGTGCATCTGGATT-3′	

nt: nucleotide.

All amplifications were conducted in a CFX Real-Time PCR System (Bio-Rad Laboratories Inc., Richmond, CA, USA) with an initial incubation period of 7 min at 94 °C, followed by 40 cycles at 95 °C for 5 s (denaturation), 55 °C for 30 s (annealing, amplification and acquisition of fluorescence) and 72 °C for 10 s (extension). Finally, a dissociation curve from 60 °C to 94 °C with an increase interval of 0.5 °C was performed.

Each qPCR run included a negative control, a positive control, and a separate reaction for Actin DNA copies as an internal control (IC). Each sample was performed in triplicate and a sample was considered positive if at least two of the triplicates were positive with both pairs of primers. For a result to be considered positive, the threshold cycle (*Ct*) had to be lower than 38, as determined by a standard curve for the set of ToxoRoc primers, according to previously described methods (Gracia et al., 2021).

### 2.4 Statistical analyses

To calculate the minimum sample size required to obtain significant results, WinEpi 2.0 (http://www.winepi.net/winepi2/ accessed on 5 July 2019) (Vallejo et al., 2013) with the “estimate proportion (random sampling & perfect diagnostic)” option was used. As no data have been previously published for Spain, the estimated proportion we used was that obtained in Europe by Belluco et al. (2016). Therefore, in order to calculate an estimated ratio of 9% with an accepted error (or precision) of 5% and a confidence level of 95%, the minimum sample size was 126 individuals.

All qPCR results were registered in an Excel file and prevalence was expressed as a percentage with a 95% confidence interval. For this purpose, free software obtained through “VassarStats: Website for Statistical Computation” was used (http://vassarstats.net/).

## 3 Results

All 216 samples had satisfactory internal control amplifications; positive control amplified correctly in each run. As previously observed (Gracia et al., 2021), the sensitivities of both sets of primers, ToxoRoc and ToxoRepeat, for the 529 bp repeat element were similar. Parasite DNA amplification was observed in 53/216 samples and no amplification was observed in 163/216 samples. Therefore, the prevalence of *T. gondii* in meat observed in this study was 24.5% (95%, CI = 19–30). The mean *Ct* value obtained from positive samples was: 31.18 (SD = 1.88) (Table 2).

**Table 2:** Real-time PCR positive results for muscle samples tested in this study.

*Ct* intervals	Number positive	*Ct* mean	SD
26–28.99	9	28.44	0.83
29–30.99	16	29.97	0.56
31–32.99	18	32.30	0.45
33–34	10	33.59	0.22

*Ct*: Cycle threshold.

## 4 Discussion

The prevalence of *T. gondii* assessed in this study was 24.5% with a total of 53 positive samples. To the best of our knowledge, this is the first study to determine the presence of *T. gondii* in sheep meat in Spain, specifically in the supply area of a slaughterhouse located in Ciudad Real, Castilla-La Mancha. The number of animals sampled (*n* = 216) lay above the minimum required number (*n* = 126), This leads us to consider these results as representative of the sheep population in the center, west, and south of Spain.

This prevalence is higher than the global prevalence (14.7%) observed in other studies performed on sheep meat, and also higher than the European average (9%) observed by Belluco et al. (2016). In molecular technique results from European regions, a lower prevalence has been observed in Switzerland (3.3%) (Berger-Schoch et al., 2011), Ireland (3.6%) (Halová et al., 2012), England (10.2%) (Mason et al., 2010), Italy (12.9%) (Gazzonis et al., 2020) and Portugal (17.6%) (Lopes et al., 2015). However, it is difficult to compare those results with ours due to differences in the types of molecular methods used.

Many of the studies mentioned above did not report the animals´ age. The differences in prevalence between our research and those studies may be due to differences among the samples in terms of age. In our study, all animals were adults, and *T. gondii* has a higher prevalence in that age group than in lambs, as observed in the review conducted by Dubey et al. (2020). As the study by Katzer et al. (2011) concluded, adults are more exposed to the parasite and for a more extended period of time, thereby accumulating *T. gondii* infection throughout life and confirming horizontal transmission as a significant route of infection.

The lower prevalence of *T. gondii* observed in other studies (Mason et al., 2010; Berger-Schoch et al., 2011; Halová et al., 2012; Lopes et al., 2015) could also be related to the distribution of parasite tissue. The distribution of *T. gondii* within one tissue is random, and parasite density may be low if only one sample location is studied (Glor et al., 2013). Therefore, a negative result should be interpreted with caution, as the parasite could be present in unexamined parts of tissues. For this reason, to avoid sampling error in our study, we obtained and homogenized meat from different muscular locations of each individual.

Detection of *Toxoplasma* in the current study was performed by qPCR. This method only demonstrates the presence of *T. gondii* DNA in our samples, but not the presence of viable parasites capable of triggering a human infection. We thus may have overestimated the degree of parasitization. However, a study performed by Pardini et al. (2016) on chicken meat isolated viable parasites by bioassay in proportions quite similar to those obtained by PCR. As no technological treatment was applied in our study, it can be assumed that many parasites detected by PCR were viable. However, it is also possible that, when sampling adult animals, the *Toxoplasma* cysts were calcified, thus implying a decreased risk of transmission. In order to complete molecular results, genotyping positive samples would be of interest. The mean *Ct* value obtained from positive samples was 31.18 (SD = 1.88), which made sequencing impossible.

Despite these drawbacks, the present study still supports the premise that sheep meat consumption can represent a risk factor for human health. Most consumed sheep meat (especially in Spain) is lamb meat, thus making it likely that the risk for the consumer is lower. However, it should be noted that the slaughterhouse featured in this study exports mainly to Morocco, where most consumed meat comes from adult sheep. Further studies should be conducted to assess the actual prevalence of *T. gondii* in lamb meat, which is the meat of this species most consumed in Spain.

## 5 Conclusions

This study confirms the presence of *T. gondii* DNA in mutton for the first time in Spain. These results should encourage researchers to investigate the presence and viability of *T. gondii* in lambs, aiming to assess the actual risk of infection for the Spanish human population.

## Funding

This study was supported by 10.13039/501100008530European Regional Development Fund (FEDER) / 10.13039/100014440Ministerio de Ciencia, Innovación y Universidades – 10.13039/501100011033Agencia Estatal de Investigación – Project RTC-2017-6594-2. Also, funding for the research was likewise provided by the research groups “Reproducción Asistida y Sanidad Animal (Ref: A17_17R)” and “Grupo de referencia enfermedades priónicas, vectoriales y zoonosis emergentes (Ref. A05_17R)”.

## Declaration of Competing Interest

The authors declare that they have no known competing financial interests or personal relationships that could have appeared to influence the work reported in this paper.
